# Sphingolipids and ceramides in human aqueous humor

**Published:** 2013-09-19

**Authors:** Ayman J. Aljohani, Gustavo C. Munguba, Yenifer Guerra, Richard K. Lee, Sanjoy K. Bhattacharya

**Affiliations:** Bascom Palmer Eye Institute, University of Miami, Miami, FL

## Abstract

**Purpose:**

To determine the differential profiles of sphingomyelin, sphingoid base, sphingoid base-1-phosphate and ceramide lipid species and their quantitative differences between control and glaucomatous aqueous humor (AQH) derived from human donors.

**Methods:**

AQH from control and primary open-angle glaucoma donors was collected and subjected to lipid extraction using suitable modifications of the Bligh and Dyer method. Proteins were estimated using Bradford’s method. Lipids were identified and ratiometrically quantified in a two-step process using precursor ion scan or neutral loss scan (NLS) with appropriate class-specific lipid standards on a TSQ Quantum Access Max mass spectrometer following established procedures. Primary human trabecular meshwork cells and video microscopic imaging were used to assess changes in cell shape and motility upon exposure to 20 pmol of Cer(d18:0/18:1(9Z)) in 10% dimethyl sulfoxide (vehicle).

**Results:**

We identified several species of sphingomyelin, sphingoid base, sphingoid base-1-phosphate, and ceramides that were common between control and glaucomatous AQH. Some unique lipid species in these classes were also identified in controls but not in glaucoma and vice versa. We found exposure to 20 pmol of Cer(d18:0/18:1(9Z)) resulted in changes in the trabecular meshwork cell shape and observed motility changes compared to vehicle-only control.

**Conclusions:**

Most lipids belonging to the sphingomyelin, sphingoid base, sphingoid base-1-phosphate, and ceramide species were common between control and primary open-angle glaucoma donors. However, some sphingolipids and ceramides were found to be uniquely present in control but absent in the glaucomatous AQH and vice versa. Identification of unique lipid species present or absent in the pathophysiological context may contribute further insight into glaucoma pathology.

## Introduction

Glaucomas are a group of irreversible blinding diseases that affect approximately 60 million individuals worldwide and represent a significant health burden [1], especially relative to quality of life. Primary open angle glaucoma (POAG) is one of the most prevalent forms of glaucoma. Elevated intraocular pressure (IOP) is one of the most important risk factors for glaucoma. IOP is also the major modifiable parameter that affects the progression of glaucomatous neurodegeneration of the optic nerve. Elevated IOP results mainly from decreased aqueous outflow in the anterior eye chamber. Aqueous outflow experiences most resistance at a filter-like region termed the trabecular meshwork (TM) [2]. Increased resistance to outflow occurs in the glaucomatous TM, and compositional analyses of TM tissue and TM cells have been widely conducted [3,4].

Prostaglandin analogs that were originally discovered in the iris [5] have been found to increase the aqueous outflow via the secondary uveoscleral aqueous humor (AQH) outflow pathway [6-8] and are used for lowering IOP in glaucoma patients. IOP can also be lowered by increasing aqueous outflow via the TM pathway (also known as the conventional pathway) or by decreasing aqueous production [9]. Many medications (e.g., β-blockers and carbonic anhydrase inhibitors) decrease AQH production [10]. Apart from pilocarpine, a muscarinic agonist of inferior efficacy to prostaglandins with significant side effects, no other commercial glaucoma drugs are available to enhance aqueous outflow via the TM pathway [9-11].

In contrast to the TM, compositional analyses of the AQH remain less well studied. The existence of bioactive lipids and their possible role in the regulation of conventional outflow facility has been conjectured [12]. Until recently, two limitations existed with respect to experimental methods for identifying and quantifying lipids within the AQH in addition to the limiting amounts of AQH that can be drawn from living patient samples or even obtained from cadaveric eyes. These limitations were as follows: (1) the limiting amount of total lipid material from individual donor AQH and (2) the requirement of expertise in a vast amount of chemistries (>5000 different chemistries) for the identification of approximately 9,000–100,000 lipid species that are conjectured to exist in mammalian systems [13]. These were critical barriers for high throughput lipid profiling in AQH samples. Recent advancements in mass spectrometry and commensurate developments in bioinformatic approaches and lipid databases [14-17] have eliminated these critical barriers, thereby allowing for comprehensive analyses of class-specific lipids in the tiny amounts of AQH derived from living individuals [18].

Sphingolipids (and sphingomyelin in particular) have been shown to regulate many cellular and systemic events including apoptosis, the cell cycle, cellular growth, and inflammation [19-24]. Acar and coworkers also used some of these standardized methods for the characterization of phospholipids of red blood cells and optic nerves from POAG patients [25-27]. More recently, we have used these approaches for the determination of phospholipid profiles of the TM [4]. Most compositional analyses of AQH involve proteomics [28-31], while a few involve metabolomics [32-35]. We recently reported analyses of AQH cholesterol and psychosine using a lipidomic mass spectrometry approach [36]. Other lipid classes in the AQH remain to be analyzed. With the tiny amount of biological samples, shotgun lipidomic methods have evolved which utilize direct infusion without liquid chromatography [16,18,37]. The neutral loss and precursor ion scan (PIS) parameters for triple quadrupole mass instruments have recently been standardized for analyses of all lipid classes [16,18].

In this study, we have determined the profiles of sphingolipids and ceramides present in the AQH of human control and POAG donors. Using a powerful lipidomic approach coupled with novel bioinformatic databases, we characterized the molecular signature of four major sphingolipid species present in the human AQH. Furthermore, we quantitatively compared distinct differences between glaucomatous and age-matched control eyes, identifying potential molecules for further experimentation to determine their biological role in modulating TM cell behavior.

## Methods

### Donor information and aqueous humor procurement

AQH from POAG and control human subjects (n=10 each totaling 20 samples; Appendix 1) were procured following institutional review board approved protocols and adhering to the tenets of the Declaration of Helsinki. AQH samples were stored at −80 °C until time of use. The mean age of donors was 69.5±11 years (Appendix 1) and both genders were included in these studies.

### Lipid preparation

Aqueous humor was subjected to extraction of lipids using the Bligh and Dyer method [38], as described previously [36]. Prior to extraction, a fixed amount of a standard (1,2-ditridecanoyl-sn-glycero-3-phosphocholine; catalog number 850,340; procured from Avanti Polar Lipids, Alabaster, AL; precursor ion mass of 649.89 identified in positive ion mode for a product ion m/z of 184.04 at 35 V) [39] was added to AQH to determine recovery, ensuring >99% and uniform recovery of this added standard across all samples analyzed. The organic phase with extracted lipids was dried in a Speed-Vac (Model 5301, Brinkmann Instruments Inc., Westbury, NY). Samples were regularly flushed with argon gas to prevent oxidation. The aqueous phase containing proteins was stored at −80 °C for protein quantification. All extractions and subsequent handling were carried out using glass vials, as polyvinyl plastic was completely avoided.

### Mass spectrometric analysis

A triple quadrupole electrospray mass spectrometer (TSQ Quantum Access Max; Thermo Fisher Scientific, Pittsburgh, PA) was used for analysis of lipids in infusion mode using the TSQ Tune of Xcalibur 2.3 software package. Extracted lipids were dried and resuspended in liquid chromatography–mass spectrometry (LC-MS) grade acetonitrile:isopropanol (1:1). Samples were infused with a flow rate of 10 µl/ min and analyzed for 1.00 min with a 0.500 s scan. Scans typically ranged from 200 m/z to 1,000 m/z unless specified otherwise. The full width at half maximum peak was set at 0.7 and collision gas pressure was set at 1 mTorr. Sheath gas (nitrogen) was set to 20 arbitrary units. Auxiliary gas (argon) was set to 5 arbitrary units. For analyses of the sphingomyelin, sphingoid base, and ceramide classes, the identifications were performed using neutral loss scan (NLS) for m/z 213.2, 48 and 256.2 with collision energies of 50, 18 and 32 V, respectively; except for ceramide (in negative ion mode), all other scans were carried out in positive mode. For sphingoid base-1-phosphate, PIS was performed for product ion m/z of 79.1 in negative ion mode at 24 V collision energy. The spray voltage, ion mode, and collision energies were based on previous studies [16,40]. The analytical parameters for sphingolipids and ceramides described here are based on standardized collision energy settings as suggested in the recent literature for automated shotgun lipidomics [16,18].

### Lipid and protein quantification

The quantification was performed using an internal standard for each lipid class (different from the standard for determination of extraction efficiency), N-oleoyl-D-erythro-sphingosylphosphorylcholine, D-erythro-sphingosine, D-erythro-sphingosine-1-phosphate, and N-oleoyl-D-erythro-sphingosine (catalog numbers 860,587; 860,490; 860,402; 860,519, and precursor ion masses of 729.08, 299.5, 379.47, and 563.94, respectively, procured from Avanti Polar Lipids) for sphingomyelin, sphingoid base, sphingoid base-1-phosphate, and ceramide, respectively, using two steps [16,41]. In the first step, the class-specific standard was used to quantify abundant species; in the second, the quantification of the first step was used for quantification of less abundant species in the same class [16,18]. For each of the lipid classes presented here, n=10 donors each for control and glaucomatous AQH were used for the final analyses. Class-specific lipids were quantified using specific quantitative lipid standards, as mentioned above. About 10 scans each with and without internal standard (usually in the range of 0.1–2 pmol) were performed for each sample. The scan data with the internal standard was used for quantification in a two-step process, as described above. The scan data with and without the internal standard were inspected for each dataset by two independent observers (not involved in this study) for reproducibility.

Corresponding aqueous phase–extracted proteins were subjected to protein quantification using Bradford’s method [42]. A subset of proteins samples was also subjected to densitometric quantification using a bovine serum albumin standard (amino acid quantified) after electrophoretic separation on a PHAST gel system (GE Healthcare Bio-Sciences AB, Uppsala, Sweden) [43].

### Data analysis, ratiometric quantification, and statistical analysis

Representative spectra for each sample were carefully manually inspected by two independent observers from 10 spectra collected for each sample with and without the internal standard (total 20 spectra). The spectra were manually inspected for reproducibility of peaks and overall intensity. We paid particular attention to the reproducibility of individual m/z peaks in several identical spectra. Spectra converted to netCDF files were imported into MZmine 2.9 [44], subjected to noise removal (usually E2 levels), and analyzed for peak identification using a custom database made by LIPID MAPS (LIPID MAPS Structure Database [LMSD]; Nature Lipidomics Gateway, La Jolla, CA) in a fashion similar to that for phospholipids, as described previously [39]. Two-step ratiometric quantification was achieved using the MZmine 2.9 program, as described above. Lipid concentration was normalized to protein amount estimated from the corresponding aqueous phase using Bradford’s method. The cumulative data for each of the four sphingolipid classes were further analyzed using MZmine 2.9 and Excel macros written in house [45] to determine the presence of common and unique lipid species in control and glaucomatous samples. In MZmine 2.9, an isotopic peak grouper is applied to the peaks. The isotopic peak grouper attempts to find peaks in a peak list that form an isotope pattern. Once the pattern has been found, it saves the information about the charge and isotope ratios, and additional isotopic peaks are removed from the peak list. Here, the representative isotope was the highest peak with the highest m/z to match our database.

A two-tailed *t* test was performed to compare quantities of lipids, measured by ratiometric quantification, between glaucoma and control as performed for our previous study on AQH cholesterol and psychosine [36]. Reported lipids were found statistically significant in the *t* test (p≤0.05). For lipids that were unique, a value of zero was used for the groups devoid of the specific lipid. The number of samples was then assumed to be equivalent to the frequency of occurrence of the unique group. The select common lipid species had statistically significant differences between the control and POAG, as determined by analysis of variance. Scheffe’s post hoc test showed that select lipids species in controls were statistically different from POAG (p≤0.05).

### Cell culture

Human primary TM cells were derived from six cadaver donor eyes (all Caucasian; three males, ages at death and enucleation of 38, 37, and 41 years; and three females, aged 37, 48, and 49) following previously published protocols [3]. Primary TM cells were cultured in media containing Dulbecco’s Modified Eagle Medium 1X (cat# 10–017-CM, Mediatech Inc.), 10% heat-inactivated fetal bovine serum (FBS; cat# S11150H, Atlanta Biologicals), 0.5% 200 mM L-glutamine (cat# 9057–448, HiMedia VWR), and 1% antibiotic solution (cat# 30–004-CL, Mediatech Inc.) and incubated at 37 °C in a 5% CO_2_ cell culture incubator. Further propagation was continued in the same culture medium to at most five passages. Cells were then subjected to a serum-free media adaptation. Cells were gradually weaned off of the 1X Dulbecco’s Modified Eagle Medium supplemented with 10% heat-inactivated FBS, and were washed with 1X Phosphate-Buffered Saline (PBS; cat#21–040-CV, Mediatech Inc, Manassas, VA) to remove trace lipids. They were then cultured into 1X Dulbecco’s Modified Eagle Medium supplemented with 5% heat-inactivated FBS and incubated for several days, washed with 1X PBS, transferred into serum-free media, and stabilized for 1 week before cell culture studies. The use of serum free-media was vital to the outcome to avoid contamination of endogenous lipids from the supplemented FBS. Cells were grown to >80% confluence and confirmed by observation with a 100X inverted light microscope (VWR VistaVision Radnor, PA). The powdered form of unique lipid (Table 1) Cer(d18:0/18:1(9Z); cat# 860,624 Avanti Polar Lipids, Alabaster, AL) was dissolved in a 10% solution of dimethyl sulfoxide (DMSO; cat# BDH1115–1LP, VWR) and diluted in 1X PBS for a final concentration of 20 pmol and added to a >80% confluent monolayer of primary TM cells for visualization of change in morphology. A 10% solution of dimethyl sulfoxide (DMSO) with no lipids was added to another >80% confluent monolayer of TM cells as a control and imaged simultaneously. Trypan blue assays to determine cell viability were performed using routine and established protocols and images were taken at 10X magnification.

**Table 1:** Unique lipid species in the aqueous humor.

**Sphingomyelin species**			
Lipid Species*	m/z**	Average lipid amount	Donor frequency
**Control**			
SM(d18:2/18:1)	723.8	0.004	1
**Glaucoma**			
SM(d16:1/17:0)	691.4	0.36	3
SM(d16:1/18:1)	698.0	0.16	2
**Sphingoid base species**			
**Control**			
Sphinganine	304.5	1.04	1
Glaucoma			
(4E,8Z,d18:2) sphingosine	293.4	0.68	3
**Sphingoid base -1-phosphate**			
**Glaucoma**			
C16 Sphinganine-1-phosphate	358.0	9.55	1
**Ceramide**			
**Control**			
Cer(d18:0/18:1(9Z))	569.9	1.89	2
Cer(d18:0/26:0)	683.7	137.14	2
Cer(d18:1/26:1(17Z))	680.0	55.39	3
SM(d16:1/25:0)	804.1	143.20	3
**Glaucoma**			
Cer(d18:0/24:1(15Z))	654.4	0.01	1
CerP(d18:1/22:0)	706.4	0.18	2
CerP(d18:1/24:0)	734.2	0.09	2
SM(d18:0/16:0)	709.2	0.23	2
SM(d18:0/18:1(9Z))	734.7	0.12	3
SM(d18:1/25:0)	833.2	0.20	5

*The lipid species identification is based on Lipidmaps database, used as a *.csv file for bioinformatic analyses with MZmine 2.9 program. ** A representative mass/charge ratio is presented (variations in m/z was reconciled by MZmine 2.9). Average standard non-normalized dataset is presented here. For some lipid species identified, standard deviation value could not be calculated due to lack of presence in all samples. Average lipid amount is pmol lipid per species/µg protein.

### Time-lapse imaging

Time-lapse microscopy was performed and differential interference contrast images of the cells were taken on an Axiovert 200M Microscope (Carl Zeiss, Inc., Jena, Germany) and analyzed with the accompanying software (Carl Zeiss AxioVision Rel. 4.8). Images were taken every minute for 20 min, starting 10 min after the addition of 20 pmol of the Cer(d18:0/18:1(9Z)) and control (vehicle only–10% DMSO) solution. Acquired regions of interest were matched at 5 min increments for 20 min and used for comparison of morphology. Representative images of the Trypan blue exclusion test of cell viability were used to determine the number of viable cells present in the TM cell suspension following the time-lapse imaging. A viable TM cell will have a clear cytoplasm, whereas a nonviable TM cell will have a blue or dark cytoplasm. The images were analyzed by three independent observers to give scores for the relative motility of cells, which were used to render the bar graph between 10% DMSO–only controls and cells subjected to Cer(d18:0/18:1(9Z)). No relative cell movement was scored as 0, while maximum relative cellular movement (found with exposure to 20 pmol of a phosphatidylserine species [PS(O-16:0/15:0)] under identical conditions) was taken as 10. All time-lapse images were generated at 20X magnification.

## Results

### Workflow overview

**Figure 1:**
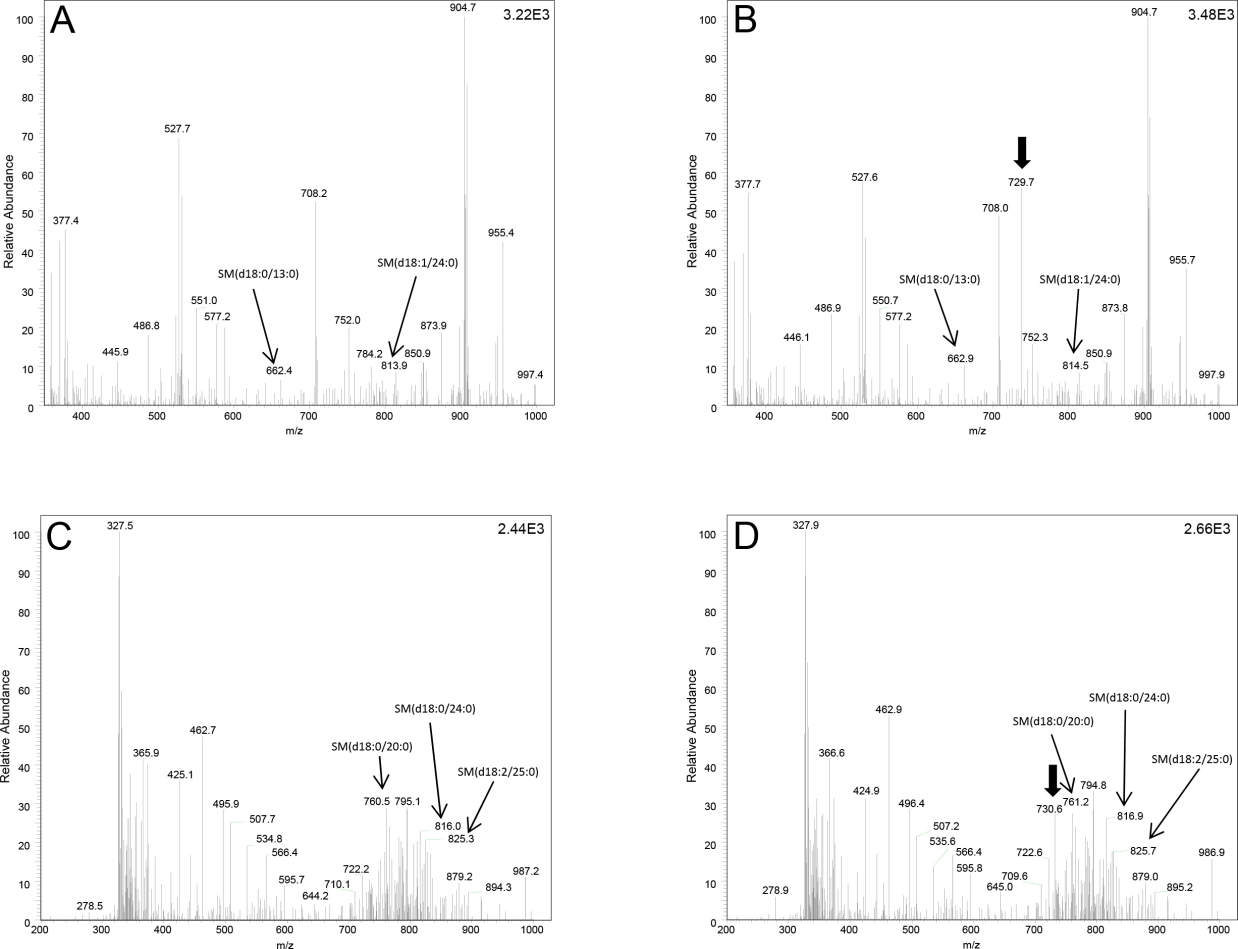
Representative electrospray ionization tandem mass spectrometric analysis of sphingomyelins extracted from control human aqueous humor in the positive-ion mode. **A**: Neutral loss scan (NLS) of m/z 213.2 corresponding to sphingomyelin class. **B**: NLS as above with internal standard addition (arrow head; m/z ratio of 729.1) enabling ratiometric quantification of all identified lipids in each sphingomyelin class. The NLS for m/z 350–1000 is shown. **C**: Precursor ion scan (PIS) of m/z 184 corresponding to choline moiety within the sphingomyelins. **D**: PIS as above with internal standard addition (arrow head; m/z ratio of 729.1), enabling ratiometric quantification of all identified lipids in sphingomyelin class using PIS. Thin arrows depict the indicated species. Appendix 2 is an enlarged version of this figure.

**Figure 2:**
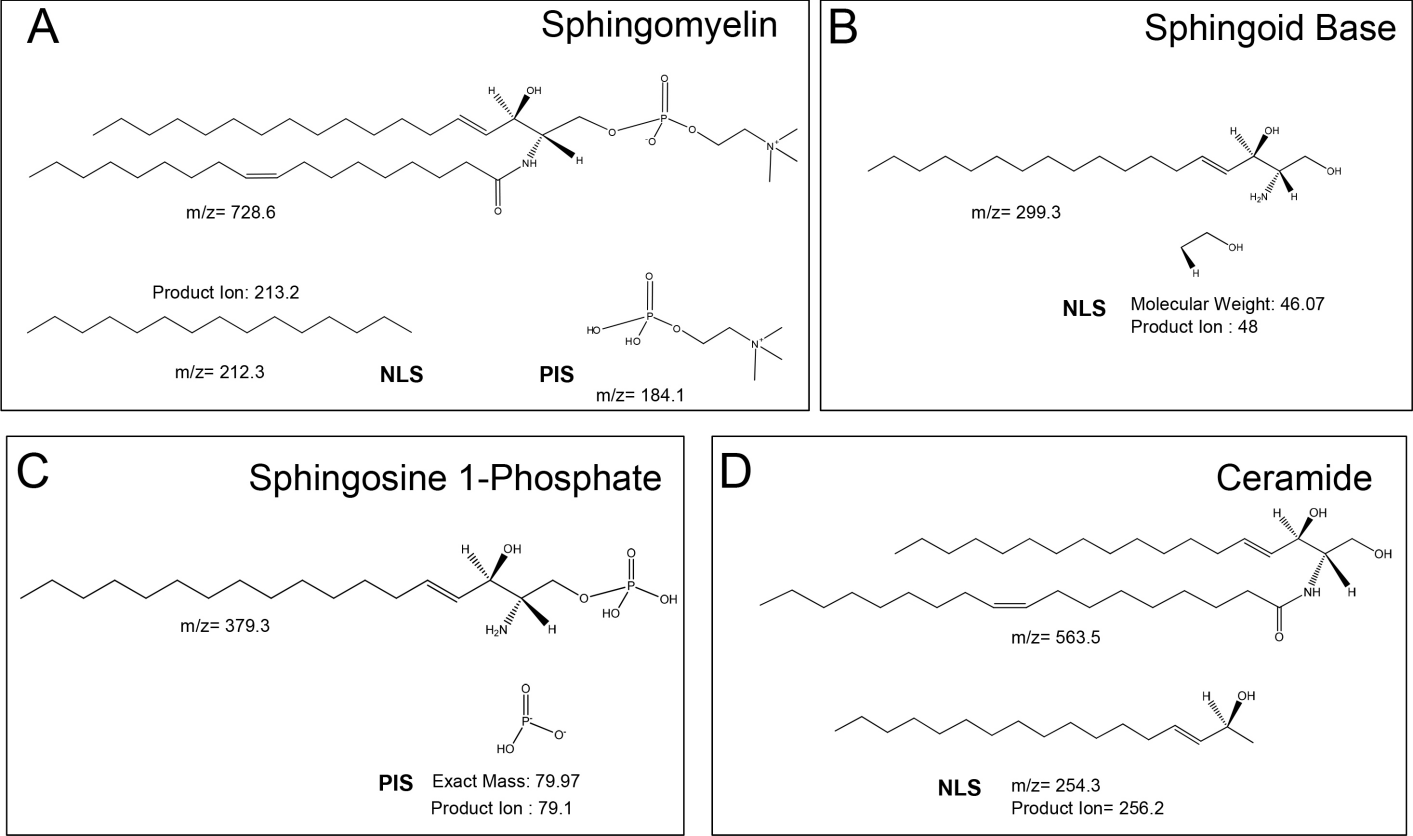
Schematic diagram of sphingolipids and ceramides showing precursor and product ions. **A**: This panel illustrates the structure of sphingomyelin standard with an m/z ratio of 728.6; the product ions can be generated in neutral loss scan (NLS) mode (m/z=212.3) or precursor ion scan (PIS) mode (m/z=184.07), as shown. **B**: The structure of the sphingoid base standard with an m/z ratio of 299.3 is depicted; the product ion is generated in NLS (product ion=48) as shown. **C**: The structure of the sphingosine-1-phosphate standard with an m/z ratio of 379.3 is depicted; the product ion is generated in PIS (product ion=79.1), as shown. **D**: The structure of the ceramide standard with an m/z ratio of 563.6 is depicted; the product ion is generated in NLS (m/z=254.3), as shown.

We analyzed the mass spectrometric profiles of sphingolipids (sphingomyelin, sphingoid base, sphingoid base-1-phosphate) and ceramides. Representative sphingomyelin scan profiles for AQH under NLS and PIS parameters are shown (Figure 1), depicting reproducible spectra in the absence and presence of a class-specific ratiometric standard. Independent spectrometric acquisition performed in either the NLS mode (Figure 1A,B) or PIS mode (Figure 1C,D) showed highly reproducible results, differing only for the additional internal standard, as expected (depicted by an arrow). For sphingomyelins, we performed analyses for a neutral loss difference of 213.2 (Figure 1A,B), which captured all sphingomyelins [18]. A schematic diagram of sphingomyelin, sphingoid base, sphingoid base-1-phosphate, and ceramide depicting the diagnostic product ions (fragment) for PIS or NLS analyses has been shown in Figure 2. However, sphingomyelins also possess a phosphocholine moiety. Analyses can be performed for the phosphocholine fragment (m/z 184), only a subset of which are sphingomyelins (Figure 1C,D); the latter was done only to validate some of the entity identified as sphingomyelin and to determine reproducibility.

### Identification of sphingomyelin species

Quantitative lipidomic analysis of AQH samples resulted in the identification of one sphingomyelin lipid, SM(d18:2/18:1), uniquely found in control patient samples, and two sphingomyelin lipids, SM(d16:1/17:0) and SM(d16:1/18:1), uniquely found in glaucoma patient samples (Table 1). A total of 19 sphingomyelin species were common to both control and glaucomatous AQH patient samples (Table 2).

**Table 2:** Common sphingomyelin species between control and glaucomatous aqueous humor.

Lipid Species*	m/z**	Control			Glaucoma			
		Average normalized lipid amount	Standard error of mean	Donor frequency	Average normalized lipid amount	Standard error of mean	Fold change	Donor frequency
SM(d18:0/13:0)	662.5	0.17	0.03	3	0.65	0.28	3.83	8
SM(d18:0/14:0)	679.8	0.07	0.02	2	0.82	0.39	11.15	3
SM(d18:0/16:0)	707.5	0.55	0.23	6	2.73	1.98	4.95	7
SM(d18:0/17:0)	721.3	0.38		1	1.43	0.63	3.71	2
SM(d18:0/18:0)	734.7	0.92		1	1.01	0.39	1.10	2
SM(d18:0/20:0)	763.0	0.31	0.11	2	0.59	0.27	1.92	3
SM(d18:0/22:0)	791.7	0.24	0.11	5	0.23	0.06	0.97	5
SM(d18:1/12:0)	643.3	0.20	0.06	3	0.28	0.10	1.39	6
SM(d18:1/14:0)	671.9	0.05	0.01	7	0.76	0.33	16.15	7
SM(d18:1/16:0)	702.4	0.09	0.04	9	1.53	0.71	16.94	8
SM(d18:1/17:0)	713.8	0.09	0.03	9	0.46	0.17	5.09	3
SM(d18:1/18:0)	727.6	0.01		1	0.74		78.75	1
SM(d18:1/18:1(9Z))	726.0	0.43		1	0.86	0.31	2.00	3
SM(d18:1/19:0)	745.4	0.50	0.35	9	0.56	0.20	1.12	7
SM(d18:1/20:0)	760.2	0.46	0.18	9	0.304	0.12	0.65	5
SM(d18:1/22:0)	788.5	0.80	0.22	8	4.754	2.25	5.91	7
SM(d18:2/14:0)	668.8	0.07	0.00	2	0.34		4.88	1
SM(d18:2/15:0)	686.2	0.06	0.01	4	0.59	0.22	10.52	4
SM(d18:2/20:1)	754.4	0.48	0.25	8	0.88	0.60	1.83	6

*The lipid species identification is based on Lipidmaps database, used as a *.csv file for bioinformatic analyses with MZmine 2.9 program. ** A representative mass/charge ratio is presented (variations in m/z was reconciled by MZmine 2.9). Average standard non-normalized dataset is presented here. For some lipid species identified, standard deviation value could not be calculated due to lack of presence in all samples. Average normalized lipid amount is pmol lipid per species/µg protein. Fold change is the ratio relative to control.

### Identification of sphingoid base species

Quantitative assessment of sphingoid bases in AQH samples resulted in the identification of two sphingoid base species. Sphinganine was found uniquely in control patient samples, while (4E,8Z,d18:2) sphingosine was found uniquely in glaucomatous patient samples (Table 1). A total of 12 sphingoid base species were common to both control and glaucomatous AQH patient samples (Table 3).

**Table 3:** Common sphingoid base species between control and glaucomatous aqueous humor.

Lipid Species*	m/z**	Control			Glaucoma			
		Average normalized lipid amount	Standard error of mean	Donor frequency	Average normalized lipid amount	Standard error of mean	Fold change	Donor frequency
(4E,6E,d14:2) sphingosine	239.6	48.79	29.22	8	0.95	0.52	0.020	10
6-hydroxysphingosine	312.3	22.90	15.67	5	0.00			1
C16 Sphinganine	277.4	54.53	36.54	9	6.31	5.85	0.116	10
C17 Sphinganine	291.7	70.48	66.93	10	1.55	0.92	0.022	10
iso (4E,15-methyl-d16:1) sphingosine	280.6	0.02		1	0.13	0.03	5.736	3
N,N,N-trimethyl-sphingosine	346.0	92.13	65.31	9	0.86	0.57	0.009	9
N,N-dimethylsphingosine	332.1	73.67	68.76	10	2.14	2.01	0.029	10
Obscuraminol A	280.2	52.84	25.33	4	0.01	0.00		3
Penaresidin A	334.0	0.37	0	1	1.73	0.67	4.627	2
Penazetidine A	373.9	1.93	1.42	7	10.99	9.06	5.705	7
Phytosphingosine	322.0	10.59	7.73	10	0.23	0.10	0.022	9
R-Dysidazirine	309.4	0.58	0.16	2	54.11		94.020	1

*The lipid species identification is based on Lipidmaps database, used as a *.csv file for bioinformatic analyses with MZmine 2.9 program. ** A representative mass/charge ratio from normal samples is presented (variations in m/z was reconciled by MZmine 2.9). Average standard normalized dataset is presented here. Lipids in this class that were identified by our approach of analyses but are not bonafide entities for mammalian systems/humans were excluded from this list. Lipids that has not been clearly determined to originate in non-mammalian species or those that are known to exist in mammals/human despite their abundant presence in plants and fungi such as Phytosphingosine (Human metabolomics database ID: HMDB04610) were retained. For some lipid species identified, standard deviation value could not be calculated due to lack of presence in all samples. Average normalized lipid amount is pmol lipid per species/µg protein. Fold change is the ratio relative to control.

### Identification of sphingoid base-1-phosphate species

Quantitative lipidomic analysis of AQH samples resulted in the identification of only one sphingoid base-1-phosphate lipid, C16 sphinganine-1-phosphate, uniquely found in glaucomatous patient samples (Table 1). A total of five sphingoid base-1-phosphate species were common to both control and glaucomatous AQH patient samples (Table 4).

**Table 4:** Common sphingoid base -1-phosphate species between control and glaucomatous aqueous humor

Lipid Species*	m/z**	Average normalized lipid amount	Standard error of mean	Donor frequency	Average normalized lipid amount	Standard error of mean	Fold change	Donor frequency
C16 Sphingosine-1-phosphate	347.8	1067.70	438.80	2	9.10	2.60	0.008	2
C17 Sphingosine-1-phosphate	366.8	511.70	270.04	3	14.50	1.30	0.03	2
C19 Sphingosine-1-phosphate	390.6	3.90		1	8.70	3.00	2.24	2
Phytosphingosine 1-phosphate	399.2	11606.60	4449.00	2	8.60		0.001	1
Sphinganine-phosphate	382.7	8268.50	5828.50	5	28.10	9.14	0.003	4

*The lipid species identification is based on Lipidmaps database, used as a *.csv file for bioinformatic analyses with MZmine 2.9 program. ** A representative mass/charge ratio is presented (variations in m/z was reconciled by MZmine 2.9). Average standard non-normalized dataset is presented here. For some lipid species identified, standard deviation value could not be calculated due to lack of presence in all samples. Average normalized lipid amount is pmol lipid per species/µg protein. Fold change is the ratio relative to control.

### Identification of ceramides

Quantitative lipidomic analysis of AQH samples resulted in the identification of four ceramide lipids uniquely present in control donor samples and six ceramide lipids uniquely found in glaucomatous patient samples (Table 1). A total of 50 ceramide species were common to both control and glaucomatous AQH donor samples (Table 5). Cer(t18:0/26:0) and SM(d18:0/15:0) were present in both control and glaucoma AQH and were statistically significantly more prevalent in control compared to glaucoma, as determined by the two-tailed *t* test (p≤0.02). Frequency and distribution of all unique sphingolipids and ceramides in donors were matched with donor information (Table 6).

**Table 5:** Common ceramide species between control and glaucomatous aqueous humor.

Lipid Species*	m/z**	Control			Glaucoma			
		Average normalized lipid amount	Standard error of mean	Donor frequency	Average normalized lipid amount	Standard error of mean	Fold Change	Donor frequency
Cer(d16:1/17:0)	524.5	3.82	2.71	10	0.18	0.09	0.05	8
Cer(d16:1/22:0)	597.4	1.90	0.64	2	0.59	0.23	0.31	2
Cer(d18:0/13:0)	497.9	10.78	8.32	10	0.19	0.06	0.02	6
Cer(d18:0/14:0)	515.7	4.14	1.83	2	0.18	0.04	0.04	4
Cer(d18:0/16:0)	540.6	4.81	2.13	2	0.16	0.03	0.03	3
Cer(d18:0/17:0)	550.6	5.71	4.68	8	0.19	0.09	0.03	9
Cer(d18:0/h17:0)	571.0	1.38	0.81	6	0.13	0.04	0.09	9
Cer(d18:0/h24:0)	670.6	2.33	0.93	7	0.09	0.02	0.04	4
Cer(d18:1/22:0)	625.9	0.22	0.10	2	0.16		0.71	1
Cer(d18:1/26:0)	675.3	0.38		1	0.16	0.05	0.42	4
Cer(d18:2/14:0)	508.5	11.52	8.85	10	0.16	0.05	0.01	10
Cer(d18:2/16:0)	533.9	31.74	36.42	10	0.15	0.05	0.005	9
Cer(d18:2/20:0)	594.9	32.88	20.17	4	0.12	0.04	0.004	2
Cer(d18:2/21:0)	606.1	14.94	10.91	10	0.15	0.06	0.01	9
Cer(d18:2/23:0)	633.5	0.70	0.25	9	0.17	0.04	0.24	7
Cer(t18:0/26:0)†	698.5	35.86	13.87	2	1.37	1.07	0.04	7
CerP(d18:0/16:0)	619.4	13.42	14.10	10	0.17	0.03	0.01	7
CerP(d18:1/12:0)	559.3	1.53	0.63	4	0.47	0.12	0.31	3
CerP(d18:1/14:0)	591.7	24.89	18.32	10	0.23	0.11	0.01	9
CerP(d18:1/24:1(15Z))	730.3	9.17	6.39	8	0.29	0.16	0.03	8
Ins-1-P-Cer(d18:1/22:0)	866.4	52.98	40.45	8	0.29	0.07	0.01	8
SM(d16:1/22:1)	760.1	33.85	26.51	10	0.26	0.14	0.01	9
SM(d16:1/24:0)	790.3	0.03	0.01	3	0.28	0.12	9.67	6
SM(d16:1/24:1)	789.5	1.46	0.17	3	0.23	0.00	0.16	1
SM(d17:1/22:0)	775.9	30.11	13.34	2	0.09	0.02	0.00	2
SM(d17:1/24:1)	802.5	12.70	10.52	9	0.23	0.06	0.02	9
SM(d18:0/12:0)	652.7	0.85	0.35	3	0.20	0.06	0.24	2
SM(d18:0/13:0)	664.4	43.74	32.97	7	0.18	0.07	0.00	10
SM(d18:0/15:0)†	694.1	3.37	0.72	2	0.26	0.15	0.08	4
SM(d18:0/17:0)	723.1	0.61	0.23	2	0.33	0.09	0.55	6
SM(d18:0/18:0)	737.6	0.01		1	0.13	0.05	50.87	2
SM(d18:0/20:0)	763.8	1.04		1	0.53		0.51	1
SM(d18:0/24:0)	821.1	3.04	1.57	3	0.16	0.06	0.05	3
SM(d18:0/26:0)	848.8	0.66	0.00	1	0.06	0.00	0.09	2
SM(d18:0/26:1(17Z))	847.4	1.53	0.40	3	0.65	0.16	0.43	2
SM(d18:1/12:0)	643.2	32.37	28.27	8	0.08	0.04	0.00	8
SM(d18:1/14:0)	678.9	4.67	1.51	3	0.14	0.02	0.03	3
SM(d18:1/15:0)	692.3	27.15	17.37	5	0.09	0.01	0.003	2
SM(d18:1/16:1)	701.2	1.52	0.54	5	0.19	0.05	0.12	4
SM(d18:1/17:0)	720.0	10.70	7.31	9	0.24	0.16	0.02	9
SM(d18:1/20:0)	763.3	182.77	81.70	2	0.18	0.06	0.001	3
SM(d18:1/24:0)	819.6	119.53	65.45	3	0.09		0.001	1
SM(d18:1/24:1(15Z))	815.6	5.34	5.07	8	0.23	0.08	0.04	9
SM(d18:1/26:1(17Z))	843.5	25.95	23.72	10	0.43	0.20	0.02	6
SM(d18:2/14:0)	673.9	39.85	32.14	6	0.30	0.11	0.01	4
SM(d18:2/15:0)	689.0	1.64	0.91	6	0.19	0.04	0.11	5
SM(d18:2/21:0)	770.3	2.99	1.71	6	0.48	0.35	0.16	9
SM(d18:2/22:1)	778.6	29.44	28.76	10	0.30	0.10	0.01	8
SM(d18:2/25:0)	831.2	12.36	10.57	7	0.58	0.22	0.05	7
SM(d19:1/18:0)	747.5	45.59	31.72	8	0.23	0.08	0.01	8

*The lipid species identification is based on Lipidmaps database, used as a *.csv file for bioinformatic analyses with MZmine 2.9 program. ** A representative mass/charge ratio is presented (variations in m/z was reconciled by MZmine 2.9). Average standard non-normalized dataset is presented here. For some lipid species identified, standard deviation value could not be calculated due to lack of presence in all samples. Average normalized lipid amount is pmol lipid per species/µg protein. Fold change is the ratio relative to control.† Cer(t18:0/26:0) and SM(d18:0/15:0) were found to have statistically significant difference between control and glaucoma using two tailed t-test (P≤0.05)

**Table 6:** Frequency and distribution of unique sphingolipids and ceramides in aqueous humor donors

Sphingomyelin											
Control aqueous humor	Donor frequency	Donor 1	Donor 2	Donor 3	Donor 4	Donor 5	Donor 6	Donor 7	Donor 8	Donor 9	Donor 10
SM(d18:2/18:1)	1										AGY049

Glaucomatous aqueous humor	Donor frequency	Donor 1	Donor 2	Donor 3	Donor 4	Donor 5	Donor 6	Donor 7	Donor 8	Donor 9	Donor 10
SM(d16:1/18:1)	2					AGY063				AGY068	
SM(d16:1/17:0)	2	AGY058					AGY064			AGY068	
Sphingoid base											

Control aqueous humor	Donor frequency	Donor 1	Donor 2	Donor 3	Donor 4	Donor 5	Donor 6	Donor 7	Donor 8	Donor 9	Donor 10
Sphinganine	1				AGY054						

Glaucomatous aqueous humor	Donor frequency	Donor 1	Donor 2	Donor 3	Donor 4	Donor 5	Donor 6	Donor 7	Donor 8	Donor 9	Donor 10
(4E,8Z,d18:2) sphingosine	3							AGY074	AGY075	AGY076	

Sphingoid base-1-phosphate											
Glaucomatous aqueous humor	Donor frequency	Donor 1	Donor 2	Donor 3	Donor 4	Donor 5	Donor 6	Donor 7	Donor 8	Donor 9	Donor 10
C16 Sphinganine 1-phosphate	1						AGY072				

Ceramides											
Control Aqueous Humor	Donor frequency	Donor 1	Donor 2	Donor 3	Donor 4	Donor 5	Donor 6	Donor 7	Donor 8	Donor 9	Donor 10
Cer(d18:0/18:1(9Z))	2	AGY051								AGY050	
Cer(d18:0/26:0)	2		AGY052				AGY056				
Cer(d18:1/26:1(17Z))	3						AGY056			AGY050	AGY047
SM(d16:1/25:0)	3						AGY056		AGY040		AGY047

Glaucomatous aqueous humor	Donor frequency	Donor 1	Donor 2	Donor 3	Donor 4	Donor 5	Donor 6	Donor 7	Donor 8	Donor 9	Donor 10
Cer(d18:0/24:1(15Z))	1	AGY067									
CerP(d18:1/24:0)	2	AGY067							AGY075		
CerP(d18:1/22:0)	2	AGY067	AGY069								
SM(d18:0/16:0)	2			AGY070					AGY075		
SM(d18:0/18:1(9Z))	3		AGY069					AGY074		AGY076	
SM(d18:1/25:0)	5	AGY067	AGY069	AGY070					AGY075		AGY057

### Effect of lipids on cell shape and motility changes

**Figure 3:**
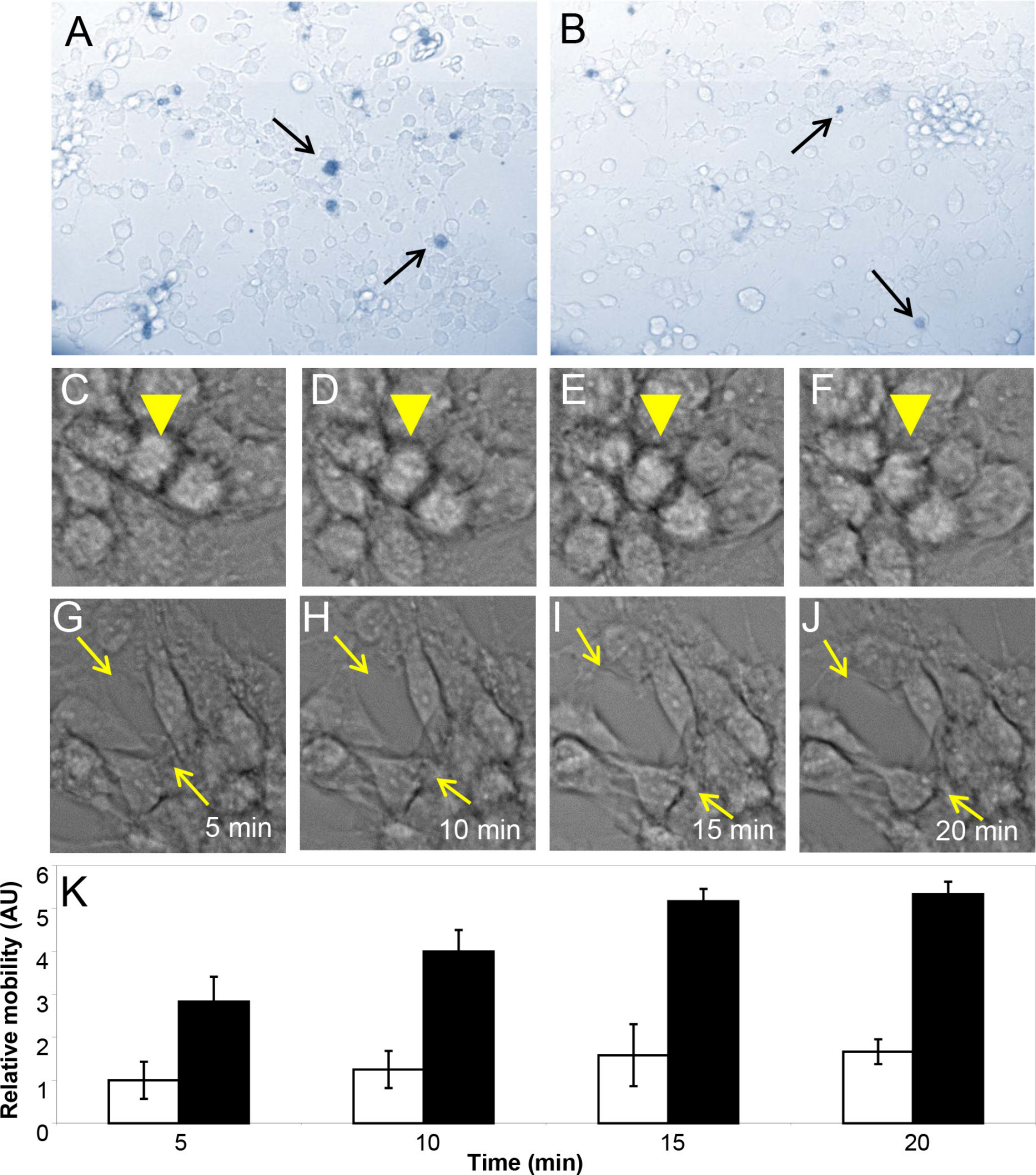
Representative changes in cell shape and relative mobility of primary trabecular meshwork cells on exposure to Cer(d18:0/18:1(9Z)). Approximately 5,000 primary trabecular meshwork (TM) cells were plated in the individuals wells of a 24 well plate and subjected to treatment with vehicle (10% dimethyl sulfoxide [DMSO]) or 20 pmol of Cer(d18:0/18:1(9Z)). Viability of cells was determined using a Trypan blue exclusion assay. **A**: Representative of control vehicle only–treated TM cells. **B**: Representative of TM cells treated with lipid in the same vehicle. **C**-**D**: Representative of control vehicle only–treated TM cells imaged between 5 and 20 min, as indicated. The arrowhead indicates a cell in the middle whose mobility is estimated relative to other surrounding cells. **E**-**J**: Representative of TM cells treated in vehicle between 5 and 20 min, as indicated. Arrows show movement of cells relative to adjacent cells. **K**: The relative mobility of cells expressed in arbitrary units sampled from five different regions of the video images. The mobility was estimated by three independent observers from the same video. Average data from 10 independent video images (Appendix 3 and Appendix 4) and standard error of the mean have been presented.

We have considered that unique lipids in the control AQH (Table 1) may have effects on TM cells. While many unique lipids identified in either group were not readily available, we found that Cer(d18:0/18:1(9Z)) could be obtained commercially. Control primary TM cells at >80% confluence showed some cell death, whether treated with nothing (data not shown) or vehicle 10% DMSO (Figure 3A); however, at 20 pmol final concentration, we did not find any appreciable cell death in treatment with Cer(d18:0/18:1(9Z)) compared to control (Figure 3B). At this concentration, the control cells showed relatively very little motility with respect to adjacent cells (Figure 3C-F), as indicated by the arrowhead in the time-lapse image, between 5 and 20 min. In contrast, the lipid-treated cells showed both changes in cell shape (Figure 3G-J) and relative motility (Figure 3 G-J, indicated by arrows and Figure 3 K). The control cells were rounder in shape (Figure 3C-F); however, upon lipid treatment, cells became elongated and spindle like (Figure 3G-J). Lipid-treated cells showed a significant difference in motility up to 3 h (data not shown). The motility difference up to 20 min after lipid treatment are shown (Appendix 3 and Appendix 4 and Figure 3 K).

## Discussion

Recent evidence emphasizes the TM as a dynamic tissue whose constant cellular motility plays a vital role in IOP homeostasis [46]. Uncontrolled elevated IOP is thought to both cause and exacerbate optic nerve damage in glaucoma-like pathology with the death of retinal ganglion cells. Control of AQH dynamics in the eye must maintain an IOP that is high enough to provide a steady supply of nutrient delivery to avascular tissues and maintain tissue rigidity for functional optics, while being sufficiently low so as to not perturb the delicate neuroretina. Tipping the balance by overproduction or the diminished clearance of AQH can result in an elevated IOP, which can lead to the irreversible loss of retinal ganglion cells. Elevated IOP as observed in POAG is generally attributed to impeded aqueous outflow as a result of increased resistance at the TM. Mechanosensing of fluid shear regulates cell shape and cell motility within the TM, thereby governing fluid flow through spaces within the TM tissue [47].

Identification of lipids in diseased and control ocular tissues is an important undertaking, as bioactive lipid involvement in mechanosensing has been described in many systems. Mechanoactivation has been shown to induce synthesis of sphingomyelin and ceramide lipid species [48,49] and is concomitant with cell shape and motility changes in yeast and other eukaryotic organisms [50,51]. Work in our laboratory has been consistent with the characterization of TM cells as dynamic regulators of aqueous flow through potassium channel subfamily K member 2 (TREK-1) mechanosensitive channels [47,52]. Importantly, TREK-1 has been implicated to play a key role in filtration in the kidney and lipids have been shown to modulate TREK-1 function [53]. The polar head groups and the acyl chain length of lipids affect the functional modulation of TREK-1, while the charge on the lipid molecule has the least effect on functional modulation. These findings are consistent with bacterial mechanosensing large channel modulation by lipids [54].

In this work, we found evidence of several ceramides and sphingolipids that are unique to the control and disease states (Table 1). The application of specific sphingolipids such as sphingosine-1-phosphate has been known to modulate AQH outflow [55]. We identified SM(d18:2/18:1), sphinganine, Cer(d18:0/18:1(9Z)), Cer(d18:0/26:0), Cer(d18:1/26:1(17Z)), and SM(d16:1/25:0) as uniquely associated with control AQH (Table 1). SM(d18:2/18:1) has been reported to be involved in neuronal function [56], but its biological role—including any role in mechanosensing—has yet to be discovered. Sphinganine, Cer(d18:0/18:1(9Z)), Cer(d18:0/26:0), Cer(d18:1/26:1(17Z)), and SM(d16:1/25:0) have been shown to affect volume-sensitive chloride current [I(Cl,swell)] in ventricular myocytes [57]. The volume-sensitive chloride current has been implicated in the regulation of TM cell shape and volume [58,59]. Thus, these sphingolipid species may possibly modulate channels responsible for the volume-sensitive chloride current or other, yet unknown, proteins. Several sphingolipid and ceramide species were also found to be unique in the AQH of POAG donors, specifically SM(d16:1/17:0), SM(d16:1/18:1), (4E,8Z,d18:2) sphingosine, C16 sphinganine-1-phosphate, Cer(d18:0/24:1(15Z)), CerP(d18:1/22:0), CerP(d18:1/24:0), SM(d18:0/16:0), SM(d18:0/18:1(9Z)), and SM(d18:1/25:0) (Table 1). Like the unique lipid entities in the controls (Table 1), the specific role of these lipids in cellular or biological function is largely unclear. As stated above, bioactive lipids and their regulation of conventional outflow facility had been conjectured [12]. We envisage that some unique lipids in the control and POAG groups (Table 1) may affect the biological activities of primary TM cells. Indeed, using >80% confluent TM cells, we found substantial changes in cell shape and motility due to treatment with Cer(d18:0/18:1(9Z)) (Figure 3G-J) compared to vehicle (10% DMSO) only–treated cells (Figure 3C-F). In these experiments, we used a concentration of 20 pmol, based on our estimates, as the prevalent concentration of Cer(d18:0/18:1(9Z)) in the normal AQH that bathes the TM. We did not find appreciable changes in cell death at this concentration (Figure 3 A, B). There is a tremendous amount of complexity with respect to age of the donor, passage of cells, medium, and culture conditions on the effect of this lipid (and a few other phospholipids that we have studied so far). We have presented representative data here, which have been found to be reproducible in primary TM cells derived from a wide variety of donors. The representative Cer(d18:0/18:1(9Z)) exposed changes in cell shape and motility that have been found reproducible in serum-free medium for a large number of different donor-derived primary TM cells.

Among the lipids that were found common between control and POAG, two—Cer(t18:0/26:0) and SM(d18:0/15:0)—exhibited a significant statistical difference (Table 5). Very limited information is available in the literature about these molecules; their potential biological role remains virtually undocumented. The databases for small molecules, such as the database and ontology of chemical entities of biological interest of the European Bioinformatics Institute, LIPID MAPS, and PubChem, show the presence of entries for Cer(t18:0/26:0) and other entities that contain this compound as their backbone. Here, it is classified as a phytoceramide; however, its involvement in any known biological pathway and its biological role remain to be elucidated. No significant upregulation in this species has been found due to induced unfolded protein response, which generally causes ceramide accumulation in both yeast and mammalian cells [60]. Mass spectrometric profiling has also revealed the presence of Cer(t18:0/26:0) in equine kidney preparations [61] and yeast [62]. SM(d18:0/15:0) is present in the European Bioinformatics Institute small molecule database and in the structure database of LIPID MAPS, but its role in biological processes have not been recorded. Recent mass spectrometric profiling has shown its presence in milk [63], and it has been suggested that it has a role in membrane fluidity, modeling, and polarity [64]. Another sphingomyelin species, SM(d18:0/18:0), was found to undergo a >50-fold change in POAG (Table 5) and was present in only a few samples (both control and POAG). These species have been detected in breast tumor models and suggested to be potentially involved in membrane remodeling [65]. Much investigation is needed before the biological role of these lipid entities is uncovered, especially in the anterior eye segment and AQH.

The foremost reason for the lack of identification and hence evaluation of endogenous lipids of normal AQH has been previous limitations of techniques such as chromatography and nuclear magnetic resonance, which have required a relatively large amount of lipids for analyses. Chromatographic techniques also have a limitation in that they necessitate detailed knowledge of the chemistry of divergent lipids for analyses. The wide variety of lipid molecules, the vast knowledge of detailed chemistry necessary for analyses, and the small quantity of samples obtained from AQH have imposed severe limitations for studies pertaining to lipid analyses from AQH. Advances in mass spectrometry and bioinformatics [13,66] in recent times (including the availability of databases) have largely surmounted many of these limitations, and researchers have taken steps toward high throughput identification and quantification of all lipid classes in the AQH [18]. Indeed, recent work using targeted lipidomics has shown promising evidence for the discovery of novel therapeutic targets in multiple sclerosis [67].

Direct infusion without liquid chromatography with or without a nanospray source have been shown to provide reproducible outcomes using shotgun lipidomics [16]. Utilization of direct infusion is especially important for analyses of tiny individual samples such as AQH [18]. Liquid chromatography in general tends to result in significant loss of lipid species before mass spectrometry in samples where tiny amounts of lipids are present, such as AQH. Hence, we have used direct infusion to analyze lipids extracted in the organic phase as described in our analyses of the phospholipids in the TM and cholesterol and psychosine in the AQH [4,36].

Although the prostaglandins are effective in lowering IOP, individuals with elevated IOP and glaucoma are often resistant to prostaglandin treatment for IOP lowering. Apart from increasing the repertoire of the molecular arsenal for IOP lowering, endogenous lipids may provide the possibility of increasing aqueous outflow via the conventional pathway (versus the uveoscleral pathway). Lowering IOP by increasing outflow via the conventional pathway may also represent a more physiological approach [7,9,68]. While further work is necessary to assess the biological consequences of many lipid species identified in the eye, in this work we have begun laying down a solid foundation for the identification and understanding of tissue- and disease-specific lipid profiles in ocular tissues.

## Appendix 1. Control and glaucomatous aqueous humor donor information and sample used for each specific lipid classes.

To access the data, click or select the words “Appendix 1.”

## Appendix 2. An enlarged image of Figure 1.

The image enables greater scrutiny to details. To access the data, click or select the words “Appendix 2.”

## Appendix 3. Video of primary trabecular meshwork cells treated with vehicle only [10% dimethyl sulfoxide (DMSO)] as control.

To access the data, click or select the words “Appendix 3.”

## Appendix 4. Video of primary trabecular meshwork cell culture treated with lipids. Primary trabecular meshwork cell were treated with 20 pmol of Cer(d18:0/18:1(9Z)) in 10% DMSO as vehicle.

To access the data, click or select the words “Appendix 4.”
